# Supplementary material for: Identification of photoperiod-induced specific miRNAs in the adrenal glands of Sunite sheep (Ovis aries)
Source: Front Vet Sci. 2022 Jul 22;9:888207. doi: 10.3389/fvets.2022.888207 (PMC9354845; doi:10.3389/fvets.2022.888207)
Supplement: Supplementary file 1 [file Data_Sheet_1.ZIP › Supplementary file/miRDeep2 file.zip!/miRDeep2 file/Know_Structure/LP42A1/oar-mir-376e.pdf]

miRBase precursor : oar-mir-376e
Total read count : 1152
oar-miR-376e-5p read count : 221
oar-miR-376e-3p read count : 710
oar-miR-376b-5p read count : 221
remaining reads : 0

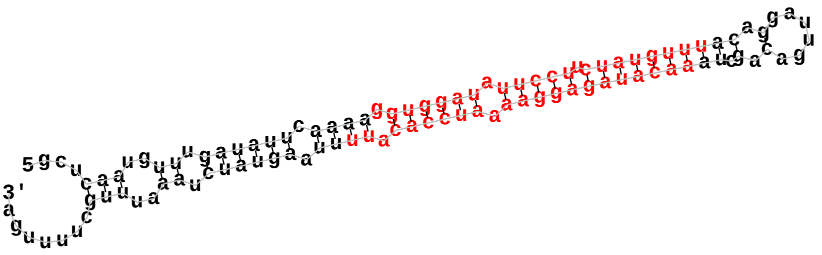

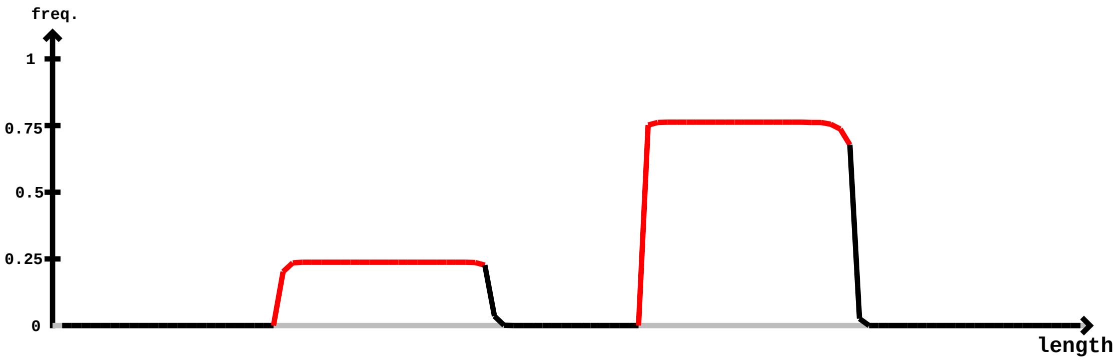


```
                                                         oar-miR-376e-3p
                      oar-miR-376b-5p
                      oar-miR-376e-5p
5'- gcucaauguuugauauucaaaagguggauauuccuucuauguuuacaggauugacagcuaaacauagaggaaaauccacauuuuaaguaucuaaaauuugcuuuuga   -3'    exp
    ((.....((((((((((.((((.((((((.(((((.((((((((((.(......).).)))))))))))))).)))))).)))).)))))).)))).)).......    reads   mm   sample
    ......................gguggauauuccuucuaugu.................................................................    1       0    seq
    ......................gguggauauuccuucuauguu................................................................    7       0    seq
    ......................gguggauauuccuucuauguuu...............................................................    154     0    seq
    ......................gguggauauuccuucuauguuua..............................................................    25      0    seq
    ......................gguggauauuccuucuauguuuac.............................................................    1       0    seq
    .......................guggauauuccuucuauguu................................................................    2       0    seq
    .......................guggauauuccuucuauguuu...............................................................    23      0    seq
    .......................guggauauuccuucuauguuua..............................................................    6       0    seq
    ........................uggauauuccuucuauguuu...............................................................    1       0    seq
    ........................uggauauuccuucuauguuua..............................................................    1       0    seq
    ............................................................aacauagaggaaaaucc..............................    1       0    seq
    ............................................................aacauagaggaaaaCccac............................    1       1    seq
    ............................................................aacauagaggaaaauccac............................    5       0    seq
    ............................................................aacauagaggaaaauccaca...........................    16      0    seq
    ............................................................aacauagaggaaaauccacau..........................    55      0    seq
    ............................................................aacauagaggaaaauccacauu.........................    602     0    seq
    ............................................................aacauagaggaaaauccacauuu........................    20      0    seq
    .............................................................acauagaggaaaauccaca...........................    1       0    seq
    .............................................................acauagaggaaaauccacauu.........................    4       0    seq
    .............................................................acauagaggaaaauccacauuu........................    4       0    seq
    ..............................................................cauagaggaaaauccacauu.........................    1       0    seq
```
